# The First Hand Transplantation in Iran

**Published:** 2013-08-01

**Authors:** A. Kalantar-Hormozi, F. Firoozi, M. Yavari, E. Arasteh, K. Najafizadeh, F. Rashid-Farokhi

**Affiliations:** 1 *Departments of Plastic Surgery, Medicine Shahid Beheshti University of Medical Sciences, Tehran, Iran.*; 2 *Departments of Hand Surgery, Medicine Shahid Beheshti University of Medical Sciences, Tehran, Iran.*; 3 *Departments of Pulmonology and Critical Care , Medicine Shahid Beheshti University of Medical Sciences, Tehran, Iran. *; 4 *Departments of Nephrology, Medicine Shahid Beheshti University of Medical Sciences, Tehran, Iran.*

**Keywords:** Hand, hand transplant, Iran, composite tissue allotransplantation, allotransplant, forearm amputation

## Abstract

Nowadays, hand transplantation is a very challenging procedure for surgeons and researchers worldwide. Despite many problems that may occur after this surgery, some centers continue to practice this highly sophisticated procedure. Herein, we report on a 38-year-old man who received hand transplant from a 24-year-old brain-dead man. This patient had lost his right hand from the lower one-third of forearm six years before after a trauma from a mincing machine. Team members organized pre-operative research, cadaver dissection, legal consultation, religious permission and discussion to patient. This procedure was done by 15 Khordad Plastic Surgery Transplant team on February 27, 2013 for the first time in Iran.

## INTRODUCTION

The first documented attempt of hand transplantation happened in 1964 in Ecuador. The transplant was rejected two weeks later because of improper immunosuppressive therapy [1]. The first successful hand transplantation took place in France in 1998; however, because of non-compliance, the patient’s hand was amputated 27 months later [2, 3]. The first double hand transplantation was performed in 2000 in France [4]. By the beginning of 2010, more than 50 hand transplantations were done round the globe (22 single and 14 double hands). Thereafter, hand transplantation became a reality [5].

Hand transplantation increases the hope in patients who lost one or both of their hands during work, war or occupational accidents. Although hand transplantation can improve the quality of life, it is not life-saving like solid organ transplantations.

Among various composite tissue allotransplantations (CTAs), hand transplantation is widely accepted and investigated [6]. After the first successful hand transplantation by Dubernard and his colleagues in 1998 [2], many centers tried to use this new technique. Although the final result was different from center to center, the main factor for achieving an acceptable outcome was found to be the patient compliance and motivation [7]. Therefore, we present the first hand transplant in Iran. 

## CASE REPORT

**Figure 1:**
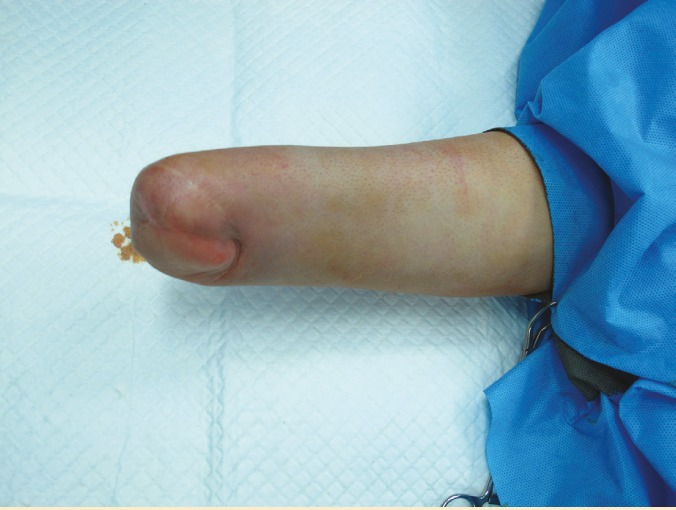
Forearm stump before hand transplant

A 38-year-old man had an accident with a mincing machine six years before and lost his right hand several centimeters above the wrist (Fig 1). After full recovery from amputation surgery, the patient was eager for a hand transplant.

On February 27, 2013 parents of a 24-year-old brain-dead man authorized the transplant team to harvest the patient’s right hand. Early morning, simultaneous with hand harvesting from the donor at Masih Daneshvary Hospital, surgery on the stump of the recipient was started at 15 Khordad Plastic Surgery Hospital. This operation took 7.5 hours.

At first, the two forearm bones were fixed to donor’s hand by two heavy plates. After bone fixation, muscles, arteries and veins of patient’s hand were coapted to the transplanted hand. Immediately after arterial repair, bleeding started from transplanted tissues. Two days later, nerve repair was performed.

**Figure 2:**
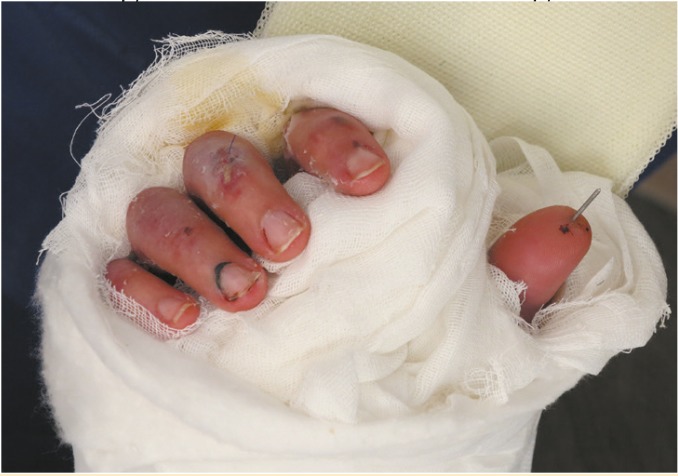
20 days after hand transplant

**Figure 3:**
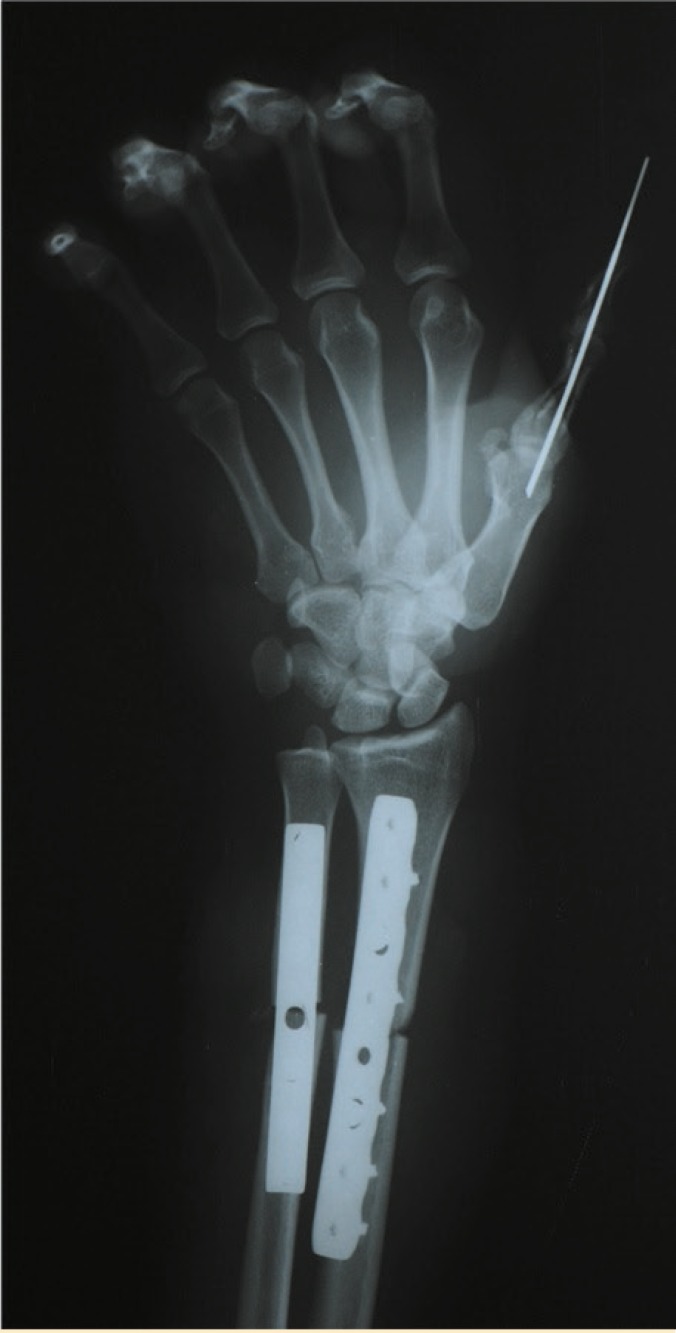
Radiogtaphy of transplanted hand 40 days after surgery

Immunosuppressive therapy was started before transplantation and continued after the surgery. After two weeks tissue biopsy showed no criteria for rejection. Dressing changed daily by the surgery team members and the patient was visited every other day by the team including medical staff for the management of immunosuppressive therapy (Fig 2). After 40 days, radiography showed well alignment of the bones (Fig 3).

## DISCUSSION

CTA is an option introduced for reconstruction of major defects. After presenting a successful hand, muscle, knee, nerve, larynx, abdominal wall and face transplantation, CTA has become one of the procedures used by plastic surgeons [8]. For this reason, during the last decade, plastic and hand surgeons paid their attention to the very challenging hand transplant surgery [9]. Although different opinions exist towards hand transplantation, there is no cessation in doing this procedure worldwide [7]. In comparison to solid organ transplantation, hand transplantation as a CTA contains more than one tissue (*e.g.*, skin, muscle, bone marrow, lymph nodes, nerve, tendon, *etc*).

Apart from jurisprudence and religious issues that were solved in Iran, hand transplantation is a very simple procedure for an experienced plastic and microvascular surgeon. On the other hand, because the transplanted organ is exposed, graft-take survey and follow-up is much easier for surgeons. In addition, care givers can readily assess the graft condition and manipulating immunosuppressive medications.

In conclusion, performing the first short-term successful hand transplant in Iran, plastic surgery transplant team is now more familiar with the procedure, complications and immunosuppressive medications for composite tissue transplantation.

Although, we have time to conclude the outcome of the first CTA in Iran, in short-term we can extend our experience to other centers that are potentially ready to replicate this procedure [9]. This should be of interest to practicing plastic surgeons because in near future, CTA may become a routine part of their practice.

Finally, we believe the need to increase our team members and make a national CTA team for research and decision-making around this young and interesting subject in order to share our experience and result to other academic centers around the world.

## Notes

**FINANCIAL SUPPORT:** None
